# Sense of community index scale: Translation, cultural adaptation, and validation of the Chinese version – a cross-sectional study

**DOI:** 10.1097/MD.0000000000045028

**Published:** 2026-01-16

**Authors:** Ling Wang, Wei Xiang, Chengxiang Liu, Ping Sun, Qiao Xu, Hui Xie

**Affiliations:** a Department of Neurosurgery, The 902nd Hospital of the Joint Logistics Support Force of the Chinese People’s Liberation Army, Bengbu, China; b Department of Cardiology and Nephrology, The 902nd Hospital of the Joint Logistics Support Force of the Chinese People’s Liberation Army, Bengbu, China; c Department of Anesthesiology, The Second Affiliated Hospital of Anhui Medical University, Hefei, China; d Department of Nursing, The 902nd Hospital of the Joint Logistics Support Force of the Chinese People’s Liberation Army, Bengbu, China; e School of Nursing, Bengbu Medical university, Bengbu, China.

**Keywords:** community awareness index scale, reliability, validity

## Abstract

This study translates and adapts the English version of the sense of community index (SCI) into Chinese and evaluates its reliability and validity. The Chinese version of the SCI was created through translation and revision. A convenience sampling method was used to select 351 elderly community residents, who completed the reliability and validity assessment of the scale. The Chinese version of the SCI includes 16 items. Principal component analysis with orthogonal rotation extracted 4 factors, accounting for a cumulative variance of 66.619%. Confirmatory factor analysis demonstrated that the chi-square/degree of freedom ratio (CMIN/DF) was 1.644, the goodness-of-fit index was 0.902, the comparative fit index was 0.941, the incremental fit index was 0.942, the Tucker-Lewis index was 0.926, the root mean square error of approximation was 0.060, and the root mean residual was 0.040. Standardized regression coefficients ranged from 0.542 to 0.798. The composite reliability for each dimension ranged from 0.726 to 0.825, while the average variance extracted ranged from 0.401 to 0.541. The Cronbach α coefficient for the entire scale was 0.854, with a Guttman split-half reliability of 0.811. The Cronbach α coefficients for individual dimensions ranged from 0.767 to 0.852. The revised Community Awareness Index Scale exhibits strong reliability and validity, making it a reliable instrument for assessing and researching community awareness among elderly individuals in community settings.

**Implications for practice**

The Chinese version of the sense of community index (SCI) provides a crucial foundation for gaining an in-depth understanding of community awareness levels among older adults. The establishment of this scientific assessment tool holds significant value for community workers and researchers in developing targeted intervention strategies. These strategies aim to enhance community awareness, encourage active participation of older adults in community activities, foster community development, and promote active aging.

## 1. Introduction

Community awareness is a fundamental concept in community psychology, often serving as a primary criterion for evaluating the effectiveness of community work. It represents both the essence and the core of community development. In the context of urban community development in China, a key priority is to enhance residents’ quality of life within the community, increase their sense of satisfaction with community living, foster a stronger sense of community awareness, and thereby promote the holistic advancement of the community.^[1]^ The strengthening of community awareness has been shown to facilitate residents’ participation in community activities^[2]^ and is positively correlated with various health outcomes and well-being indicators, including life satisfaction,^[3]^ self-reported health status,^[4]^ and quality of life.^[5]^ Accelerating urbanization and population aging have propelled the development of community-based elderly care as an innovative model. In this model, actively engaging the elderly in community governance has become an effective approach to addressing issues related to elderly care and health. Community consciousness is a critical determinant in evaluating participation in community governance. Scientifically and effectively assessing the community consciousness of the elderly, as well as fostering and developing their sense of community, can enhance their self-worth, increase their proactive engagement, and encourage active involvement in community building. This is a key factor influencing successful aging.^[6]^ Community consciousness serves as both the foundational element and a pivotal determinant of success in community nursing. By assessing the level of community consciousness among older adults, healthcare professionals can more effectively facilitate the promotion of health behaviors, enhance community cohesion, improve service utilization, advance health equity, support health monitoring and evaluation, and foster interdisciplinary collaboration. These measures collectively contribute to the establishment of a healthier and more harmonious community environment.^[7,8]^ However, due to the ambiguous nature of the concept of community awareness and the lack of widely recognized research tools, the promotion of community awareness in China has been hindered.

In recent years, the review of community awareness research by domestic scholars,^[9,10]^ along with studies describing the current status of community awareness in various populations,^[1,11]^ has led to increasing attention to this concept among Chinese researchers, making it a focal point in the field of community psychology. Domestic scholars regard the sense of community as a “spiritual union” formed through shared living experiences. By enhancing the sense of belonging, identity, emotional connection, and stability among members and between individuals and the collective, it reduces the “social distance” between people. This helps address the social risks and challenges posed by rapid urbanization and frequent population mobility in modern society. It encompasses 4 key characteristics: community identity, community attachment, community cohesion, and community satisfaction.^[12]^ Foreign scholars conceptualize the sense of community as a multidimensional construct encompassing collective efficacy and interpersonal relationships within a neighborhood. It is characterized by the emotional experiences of shared values, mutual support, and cooperation among residents, while emphasizing the sense of responsibility and collective emotional bonds among community members. This concept reflects the interconnectedness of social cohesion, communal identity, and the collaborative spirit inherent in community life.^[13,14]^ At present, the most influential and widely accepted definition is that of McMillan and Chavis,^[15]^ who conceptualize community awareness as the sense of belonging among community members, the perception of interdependent relationships, the recognition of the community’s significance, and the shared belief in collective responsibilities that address the needs of members. McMillan and Chavis proposed that community awareness can be understood through 4 dimensions: membership, influence, emotional connection, and the fulfillment of needs. Membership refers to the sense of belonging or the feeling of being part of interpersonal relationships within the community. Influence is a sense of significance, where community members feel that they are important within the community or that they can make a difference, and the community is also significant to them. Emotional connection refers to the sense of being rooted in a shared history, place, or experience. The fulfillment of needs refers to the belief that the community will meet the needs of its members.

Building upon the foundational “Four Elements” model, researchers have devised a suite of scales to quantify community consciousness, notably the SCI, BSCI, and BSCS.^[16–18]^ The SCI scale, in particular, has emerged as the preeminent instrument for assessing community consciousness and has garnered extensive utilization in international contexts.^[19,20]^ Nonetheless, given that community consciousness is inherently a psychological construct as perceived by community denizens, it is susceptible to the influences of specific temporal, spatial, and cultural social contextual variables. As such, the SCI has exhibited certain limitations in its application, including dimensions that are excessively abstract and a paucity of specificity, thereby compromising the fidelity of empirical measurements. In light of these considerations, the present study endeavors to introduce the SCI and undertake its sinicization. Owing to the divergence in cultural milieus between China and other nations, certain items on the scale may engender ambiguity when rendered in Chinese, thus necessitating a cross-cultural adaptation post-sinicization to forge a community consciousness assessment tool that is congruent with the local context. The findings of the measurement are delineated herein.^[21,22]^

## 2. Methods

### 2.1. Design and participants

All participants in this study were recruited from a single administrative community in Bengbu City, China. This community encompasses a mixed residential environment, including high-rise apartment complexes, older low-rise housing, and a small proportion of affordable housing units. Although participants shared the same community governance system and public facilities, their living environments differed in terms of housing type, building age, and surrounding amenities. These diversities enabled the inclusion of elderly residents from different socioeconomic backgrounds within the same geographic area. This study employed a convenience sampling method, distributing a total of 370 questionnaires, and ultimately recovering 351 valid responses, resulting in an effective response rate of 94.86%. Inclusion criteria: Age ≥60 years; long-term residents of the community (residence duration ≥1 year). Exclusion criteria: elderly individuals with speech or hearing impairments, mental disorders, or severe organic diseases; elderly individuals who refused to participate after thorough explanation. This study was approved by the institutional ethics committee, and all participants provided written informed consent.

### 2.2. Instruments

#### 2.2.1. General demographic characteristics questionnaire

The questionnaire was developed by the researchers and primarily encompassed demographic factors such as age, gender, ethnicity, religious affiliation, marital status, education level, living conditions, number of children, housing type, duration of residence in the community, previous occupation, and per capita family income.

#### 2.2.2. Sense of community index scale

The SCI Scale, developed by Perkins et al in 1990,^[23]^ is one of the most widely recognized and utilized instruments for assessing community awareness. The scale comprises 4 dimensions and 12 items: Membership (3 items), Emotional Connection (3 items), Influence (3 items), and Needs Fulfillment (3 items). Items 2, 6, 8, and 11 are reverse-scored. A 5-point Likert scale is employed for responses, ranging from “Strongly Disagree” (1 point) to “Strongly Agree” (5 points). The total score of the scale ranges from 12 to 60, with higher scores indicating a stronger sense of community awareness. Since its development, the scale has undergone multiple translations and applications, demonstrating robust reliability, with a Cronbach α coefficient ranging from 0.7 to 0.8.

### 2.3. Procedure

#### 2.3.1. Scale translation procedure

With the approval of the original author, Professor Perkins, authorization for the Chinese version of the scale was obtained for SCI publication, and the English version was provided by him. The translation process adhered to Brislin cross-cultural translation methodology, which involved both direct translation and back-translation of the scale_._^[24]^ Direct translation: the English version of the Community Awareness Index Scale was translated by 3 bilingual nursing lecturers and 1 university English instructor. After thorough discussions and revisions within the research team, a coordinated Chinese version of the scale was developed. In cases of discrepancies in the translation, the research team engaged in consultations with the 4 translators to reach a consensus, resulting in the final direct translation version. Back-translation: The direct translation of the Chinese version was back-translated into English by 2 researchers, both proficient in British English and with academic experience in Australia. Any discrepancies between the back-translated version and the original English scale were systematically addressed through discussions between the research team and the 2 translators. Following these deliberations, a consensus was reached, and the final back-translated version of the scale was established. The back-translated version of the scale was forwarded to Professor Perkins, the original author, via email for verification. He was invited to review the translation to ensure consistency with the concepts, constructs, and underlying principles of the original SCI scale. Following Professor Perkins’ suggestions and further discussions within the research team, the preliminary draft of the Chinese version was refined and finalized.

A nursing education professor, a PhD in statistics, a medical doctor, and 2 community nursing lecturers conducted cultural adaptation and revision of the scale’s items. With a comprehensive understanding of the concept of community awareness and the meanings of each dimension, the expert panel recommended the removal of items with low relevance to the corresponding dimensions. These included the item “The values of people in the community differ” under the Needs Fulfillment dimension, “Residents are able to solve problems within their community” under the Influence dimension, and “I care about my neighbors’ opinions of my behavior” under the Membership dimension. In consideration of the Chinese cultural context and a review of relevant literature,^[9,25,26]^ the expert panel proposed the inclusion of the following items: under the Membership dimension, “Engaging with community residents brings me joy”; and under the Influence dimension, the items “I have a certain level of influence over what happens in the community,” “People in this community have a strong mutual influence,” and “Community-organized activities significantly contribute to my personal development.” In accordance with the specific context and linguistic conventions of China, the preliminary draft of the Chinese version of the scale underwent a thorough review and refinement. The item “Very few of my neighbors know me” was revised to “Most of my neighbors know me” to better align with cultural expectations. The item “I have no influence in the community” was modified to “I have some influence on the development of the community” to reflect a more balanced and contextually relevant perception. Similarly, the item “I do not get along well with my neighbors” was adjusted to “I get along relatively well with my neighbors” to reduce the use of negative or potentially disengaging language, which could increase complexity, particularly for elderly individuals or those with lower educational levels, thereby minimizing the risk of response bias.^[5]^ Following these adjustments, the first revision of the Chinese scale was finalized. To ensure the appropriateness of the language, the accuracy of understanding, and the acceptability of the scale, 20 elderly individuals with at least 1 year of residence in the community were selected as interview subjects. Among them, 55% had an educational level of junior high school or below. The researchers administered the questionnaires, recording respondents’ age, gender, and the time taken to complete the survey. Upon completion of the questionnaires, individual follow-up discussions were conducted with each respondent to investigate the following aspects: the perceived difficulty of understanding the questionnaire; the appropriateness of the item wording in the context of Chinese culture; the alignment between respondents’ interpretations of the items and the intended constructs of the scale. Furthermore, respondents were invited to provide feedback on any items they found ambiguous or unclear, offering their personal insights to help clarify potential sources of misunderstanding. During the interview process, the researchers systematically recorded and summarized the feedback. In response to consistently reported issues, revisions and proofreading were conducted on the initial draft (revision 1) of the scale. One item in the Needs Fulfillment dimension, “Neighbors and I have common needs for the community,” was found to be overly abstract and difficult for respondents to comprehend. Based on this feedback, the item was revised to be more specific. In the course of the interviews, the majority of elderly respondents indicated that their primary community needs were focused on 4 key areas: community environment, service facilities, community safety, and the work of the neighborhood committee. This finding is consistent with previous domestic studies.^[27]^ As a result, the Needs Fulfillment dimension was refined to include 4 more concrete items: “The public environment and green spaces in the community meet my needs,” “The community service facilities meet my needs,” “I find the community security satisfactory,” and “I approve of the work of the neighborhood committee.” This revision culminated in the second version of the Chinese scale.

#### 2.3.2. Data collection procedure

A convenience sampling method was employed to select 40 elderly individuals from a community in a city in China, who met the inclusion and exclusion criteria, for a pilot study. All participants demonstrated an understanding of the item content, and the time required to complete the scale ranged from 3 to 8 minutes. Subsequently, a formal survey was conducted using the same convenience sampling method. Convenience sampling was used, and 370 elderly residents from a city in China were selected as the research subjects for the formal survey. Three investigators underwent standardized training on the research methodology, the dimensions of the scale, and the interpretation of each item. The survey was conducted in person and anonymously, with the researchers administering the questionnaire by reading each item aloud to the respondents and recording their responses. The investigators ensured strict confidentiality of the participants’ personal information. Prior to the collection of completed questionnaires, each one was carefully reviewed and cross-checked for completeness and accuracy. Any missing or inconsistent responses were promptly identified and rectified, with necessary items being supplemented or verified as needed.

#### 2.3.3. Data analysis

Statistical analysis was conducted using SPSS 23.0 (Chicago) and R version 4.3.1. Descriptive statistics for the general demographic data were presented as composition ratios, and critical ratio and homogeneity tests were employed to assess the appropriateness of the scale items. The internal consistency of the scale was evaluated using Cronbach α coefficient and Guttman split-half reliability coefficient. Content validity was assessed through the calculation of the content validity index (CVI). To evaluate the structural validity of the scale, the returned questionnaires were randomly divided into 2 groups, with sample sizes of 171 and 180, respectively. Exploratory factor analysis (EFA) and confirmatory factor analysis (CFA) were performed on these samples.

#### 2.3.4. Ethical approval

At the beginning of the questionnaire for this study, the purpose and significance of this study were introduced to the participants in writing. All the elderly people who participated in this study did so voluntarily and signed the informed consent forms. Throughout the process of completing the questionnaires, anonymity was strictly ensured. In addition, this study has received approval from the Ethics Committee of the author’s affiliated institution (Approval Number: 2024-024).

## 3. Results

### 3.1. Demographic information

A total of 351 elderly residents from the surveyed community participated in this study, including 221 males (63.0%) and 130 females (37.0%). The average age was (73.91 ± 6.0) years. Among the participants, 243 (69.2%) were married, 145 (41.3%) had a middle school education, and 170 (48.4%) had previously worked as industrial workers. Regarding residential characteristics, 57.5% of participants lived in privately owned high-rise apartments, 29.9% in older low-rise housing owned by relatives, 6.6% in affordable housing provided by the government, and 6.0% in rental units. Although they were from the same administrative community, the participants represented a diverse range of housing conditions and neighborhood micro-environments (details in Table 1).

**Table 1:** General demography data (n = 351).

Factors	Group	N (%)
Gender	Male	221 (63.0)
	Female	130 (37.0)
Age	60~	83 (23.6)
	70~	202 (57.5)
	80~	66 (18.8)
Ethnicity	Han	336 (95.7)
	Other	15 (4.3)
Religious belief	None	320 (91.2)
	Have	31 (8.8)
Marital status	Married	243 (69.2)
	Widowed	101 (28.8)
	Other	7 (2.0)
Education level	No formal education	25 (7.1)
	Primary school	78 (22.2)
	Junior high school	145 (41.3)
	High school/Secondary	72 (20.5)
	College/University	31 (8.8)
Living situation	Live alone	65 (18.5)
	Live with spouse	187 (53.3)
	Live with children	60 (17.1)
	Other	39 (11.1)
Number of children	1	32 (9.1)
	2	96 (27.4)
	3	110 (31.3)
	≧4	113 (32.2)
Housing type	Own property	202 (57.5)
	Relatives’ property	105 (29.9)
	Rental housing	21 (6.0)
	Affordable housing	23 (6.6)
Years in community	≦10	115 (32.8)
	11~20	88 (25.1)
	21~30	76 (21.7)
	>30	72 (20.5)
Previous occupation	Government/administrative	27 (7.7)
	Teacher	28 (8.0)
	Office worker (company)	45 (12.8)
	Industrial worker	170 (48.4)
	Farmer	18 (5.1)
	Freelancer	28 (8.0)
	Other	35 (10.0)
Household per capita income	500~1000	22 (6.3)
	1001~2000	25 (7.1)
	2001~3000	208 (59.3)
	3001~4000	58 (16.5)
	4001~5000	25 (7.1)
	>5000	13 (3.7)

### 3.2. Item analysis

To assess item discrimination, the total scores of the scale were ranked in ascending order. The top 27% of scores were classified as the high-score group (coded as 1), while the bottom 27% were classified as the low-score group (coded as 2). A t-test for independent samples was performed to compare the 2 extreme groups. The results indicated that the critical ratios (CR) for all items ranged from 4.627 to 9.219, with statistically significant differences observed (*P *< .001). The correlation coefficients for the items with the total scale score ranged from 0.463 to 0.647 (Table 2), all of which were >0.4. This suggests a statistically significant and moderate to strong correlation, thereby confirming that the items demonstrate a high degree of homogeneity with the overall scale.

**Table 2:** Item analysis of the community awareness index scale.

Item	Item score (SD)	Critical ratio	Correlation coeffcient	Cronbach α if item deleted
S1	3.48 (0.795)	5.055	0.555	0.845
S2	3.35 (0.935)	6.470	0.560	0.845
S3	3.43 (0.815)	6.443	0.566	0.845
S4	3.54 (0.905)	6.120	0.568	0.845
S5	2.54 (0.818)	9.219	0.609	0.844
S6	2.74 (0.915)	8.223	0.564	0.846
S7	3.19 (0.675)	8.304	0.629	0.842
S8	3.57 (0.690)	6.529	0.548	0.846
S9	2.44 (0.691)	8.182	0.572	0.845
S10	2.85 (0.627)	8.389	0.640	0.841
S11	2.24 (0.612)	7.921	0.468	0.851
S12	2..63 (0.592)	9.813	0.647	0.840
S13	3.33 (0.952)	4.627	0.463	0.851
S14	2.85 (1.071)	7.100	0.561	0.847
S15	3.41 (1.000)	6.475	0.524	0.848
S16	3.04 (0.846)	7.649	0.562	0.845

SD = standard deviation.

### 3.3. Validity analysis

#### 3.3.1. Content validity analysis

An expert panel consisting of 1 professor of nursing education, 1 PhD in statistics, 1 PhD in medicine, and 2 lecturers in community nursing was convened to assess the relevance of each item in the scale to the concept of community awareness and its associated dimensions. The experts used a 4-point scale to rate the relevance of each item: 1 = not relevant, 2 = weakly relevant, 3 = moderately relevant, and 4 = highly relevant. Items rated with a score of 3 or 4 were considered to have strong representativeness. The content validity at both the item-level (item-level CVI, I-CVI) and the scale-level (scale-level CVI, S-CVI) was calculated based on the expert ratings. The I-CVI for each item was determined by dividing the number of experts who rated the item as 3 or 4 by the total number of experts. The S-CVI for the scale was calculated as the proportion of items rated as 3 or 4 by all experts.^[28]^ The results of this study revealed that the I-CVI for individual items ranged from 0.80 to 1.00, while the S-CVI for the scale was 0.98, indicating excellent content validity.

#### 3.3.2. Correlation analysis

The correlation coefficients between the dimensions ranged from 0.206 to 0.425, while the correlation coefficients between each dimension and the total score of the scale ranged from 0.656 to 0.751. All differences were statistically significant (*P* < .01), as shown in Table 3.

**Table 3:** Correlation coefficients (r values) between dimensions of the Chinese version of the community awareness index scale and the total score of the scale.

Dimension	Total scale	Membership	Emotional connection	Influence	Need fulfillment
Membership	0.695*	1	–	–	–
Emotional connection	0.695*	0.357*	1	–	–
Influence	0.751*	0.348*	0.425*	1	–
Need fulfillment	0.656*	0.206*	0.226*	0.354*	1

* *P* <.01.

#### 3.3.3. Exploratory factor analysis

**Figure 1.**
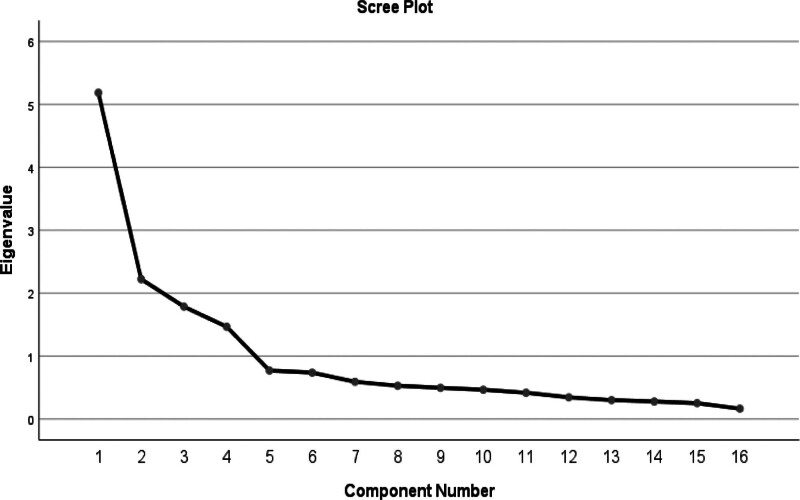
Screening plot for EFA of the Chinese version of the community awareness index scale (IBM SPSS 23.0). EFA = exploratory factor analysis.

EFA was performed using a sample of 171 elderly individuals from Chinese communities. principal component analysis with varimax rotation was employed to assess the structural validity of the scale, the appropriateness of the item composition within each dimension, and the contribution of each item to its respective dimension. The Kaiser–Meyer–Olkin measure was 0.809, and Bartlett test of sphericity yielded a significant result (χ² = 1213.161, *P* < .001), indicating that the data were suitable for factor analysis. Based on the scree plot (Fig. 1) and the factor loadings after rotation, 4 factors were extracted, each with an eigenvalue >1. The cumulative variance explained by the factors was 66.619%. All items had factor loadings >0.5, and the communalities ranged from 0.503 to 0.799, as detailed in Table 4.

**Table 4:** Exploratory factor analysis results of the chinese version of the community awareness index scale.

Dimension	Item	Factor loading matrix				
		Commo-nalities	1	2	3	4
Membership	S6. Most of my neighbors know me	0.799	**0.884**	0.053	0.115	0.091
	S5. I know most of the people in this community.	0.729	**0.832**	0.085	0.102	0.138
	S7. Interacting with community residents makes me happy.	0.723	**0.790**	0.244	0.194	0.043
	S8. I get along well with my neighbors.	0.618	**0.748**	0.218	0.060	0.084
Emotional connection	S1. I feel my community is a good place for me to live.	0.709	0.184	**0.810**	0.000	0.134
	S4. I want to live in this community for a long time.	0.669	0.134	**0.792**	0.124	0.094
	S2. Living in this community gives me a sense of home.	0.643	0.207	**0.761**	0.137	0.039
	S3. Living in this community is very important to me.	0.673	0.031	**0.735**	0.360	0.033
Influence	S9. I have a say in what happens in the community.	0.699	0.153	0.073	**0.812**	0.102
	S11. I have some influence on the development of this community.	0.629	0.008	0.118	**0.784**	-0.002
	S12. Community-organized activities significantly benefit my development.	0.624	0.200	0.179	**0.703**	0.240
	S10. People in this community influence each other well.	0.503	0.201	0.312	**0.530**	0.290
Need fulfillment	S14. The community’s services and facilities meet my needs.	0.734	0.106	0.127	0.034	**0.840**
	S13. The community’s public environment and greenery meet my needs.	0.679	-0.016	0.068	0.036	**0.820**
	S15. I feel the community is safe.	0.652	0.094	0.048	0.111	**0.793**
	S16. I approve of the work of the residents’ committee.	0.578	0.090	0.047	0.314	**0.685**
Rotated eigenvalues			2.886	2.695	2.686	2.392
Variance explained (%)			18.038	16.843	16.788	14.950
Cumulative variance explained (%)			18.038	34.881	51.669	66.619

All factor loadings were greater than 0.5, indicating that the items sufficiently represent the subscale, are shown in bold.

#### 3.3.4. Confirmatory factor analysis

**Figure 2.**
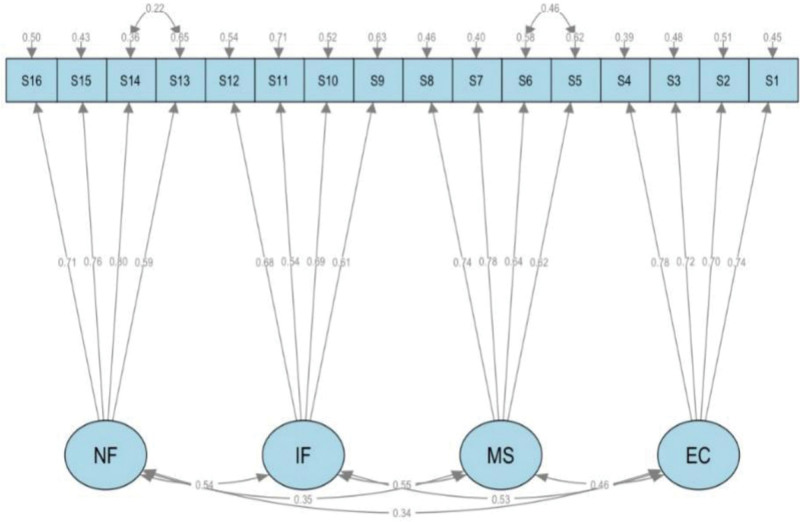
The standardized 4-factor structural equation model of the revised Chinese version of the community awareness index scale. EC = emotional connection (R version 4.3.1), IF = influence, MS = membership, NF = need fulfillment.

A CFA was performed on a sample of 180 elderly individuals from community settings using the maximum likelihood estimation method. Following model adjustments, a standardized 4-factor structural equation model was derived. The model demonstrated adequate fit, as evidenced by the following fit indices: chi-square to degrees of freedom ratio (CMIN/DF) = 1.644, goodness-of-fit index = 0.902, comparative fit index = 0.941, incremental fit index = 0.942, Tucker-Lewis index = 0.926, root mean square error of approximation = 0.060, and root mean square residual (root mean residual) = 0.040 (Fig. 2). The standardized regression coefficients for the 4 factors ranged from 0.542 to 0.798. The composite reliability (CR) for the 4 dimensions ranged from 0.726 to 0.825, while the average variance extracted (AVE) ranged from 0.401 to 0.541 (Table 5).

**Table 5:** Confirmatory factor analysis results of the chinese version of the community awareness scale.

			UNSTD	S.E.	*Z*	*P*	STD	CR	AVE
S5	<---	Membership	1	–	–	–	0.620	0.790	0.488
S6	<---	Membership	1.003	0.11	9.139	<.001	0.645		
S7	<---	Membership	0.927	0.129	7.162	<.001	0.777		
S8	<---	Membership	0.797	0.113	7.059	<.001	0.738		
S9	<---	Influence	1	–	–	–	0.610	0.726	0.401
S10	<---	Influence	0.926	0.141	6.578	<.001	0.689		
S11	<---	Influence	0.873	0.156	5.608	<.001	0.542		
S12	<---	Influence	1.028	0.157	6.529	<.001	0.680		
S1	<---	Emotional connection	1	–	–	–	0.738	0.825	0.541
S2	<---	Emotional connection	1.06	0.125	8.501	<.001	0.703		
S3	<---	Emotional connection	1.127	0.13	8.668	<.001	0.718		
S4	<---	Emotional connection	1.278	0.138	9.279	<.001	0.781		
S13	<---	Need fulfillment	1	–	–	–	0.593	0.809	0.518
S14	<---	Need fulfillment	1.456	0.179	8.157	<.001	0.798		
S15	<---	Need fulfillment	1.184	0.178	6.667	<.001	0.757		
S16	<---	Need fulfillment	1.154	0.177	6.519	<.001	0.710		

AVE = average variance extracted, CR = composite reliability, STD = standardized estimates, UNSTD = unstandardized estimates.

### 3.4. Reliability analysis

The Cronbach α coefficient of the Chinese version of the Community Awareness Index (SCI) was 0.854, with the Cronbach α coefficients for the 3 dimensions ranging from 0.767 to 0.852, as shown in Table 6. The Guttman split-half reliability coefficient of the scale was 0.811.

**Table 6:** Cronbach α coefficient test results.

Dimension	Number of items	Cronbach α coefficient
Membership	4	0.852
Emotional connection	4	0.822
Influence	4	0.767
Need fulfillment	4	0.818
Total scale	16	0.854

### 3.5. Subgroup analysis

To explore the potential influence of gender on the SCI total score, an independent samples t-test was conducted between male and female respondents. The results revealed no statistically significant difference between the 2 groups (t = 0.004, *P* = .997), suggesting that gender did not have a significant impact on the overall SCI score, as shown in Table 7.

**Table 7:** Comparison of SCI total scores by gender among elderly community residents.

Factor	Group	n	Mean ± SD	*P*
Gender	Female	130	57.38 ± 7.43	<.05
	Male	221	57.37 ± 7.65	<.05

SCI = sense of community index, SD = standard deviation.

## 4. Discussion

This study evaluated the reliability, validity, and factor structure of the revised Chinese version of the community awareness index scale (SCI) among elderly individuals in Chinese community settings. The final version of the scale comprised 16 items. Content validity and construct validity are commonly used indicators for evaluating the quality of measurement instruments.^[29,30]^ Both the item-level and scale-level content validity indices exceeded 0.8, indicating that the items in the Chinese version of the scale are highly representative and possess strong content validity.^[31,32]^ After performing CFA on the revised Chinese version of the scale, it was found that the factor analysis results differed to some extent from the original scale. The item “I hope to live in this community for a longer time,” originally belonging to the Membership dimension, and the item “I think my community is a good place for me to live,” originally belonging to the Needs Fulfillment dimension, were assigned to the Emotional Connection dimension after EFA. The item “I get along well with my neighbors,” originally belonging to the Emotional Connection dimension, was assigned to the Membership dimension after EFA. The subjects of this study were elderly people in Chinese communities, whereas the original scale was developed for community residents abroad, with a wider age range. In addition, cultural differences, diverse values, and the heterogeneity of experiences and perspectives may lead to different interpretations of dimensions such as Membership and Needs Fulfillment, resulting in inconsistencies in the scale structure. Second, in community psychology, researchers typically categorize community awareness into 2 basic types: geographic community awareness and relational community awareness. The former emphasizes the attachment and identification of community members to a specific geographic area (such as neighborhoods, communities, schools, towns, cities, etc), while the latter highlights the psychological connection of community members within a specific organization formed around shared interests or benefits (such as workgroups, hobby clubs, religious groups, online communities, etc).^[33]^ The region selected for this study was a city in China, where elderly people’s perceptions of community awareness may vary by location. Therefore, it is recommended to further test the applicability of the scale in different regions and among different populations.

To further examine the goodness of fit of the factor structure model, this study conducted CFA. The results showed that CMIN/DF <3.0, goodness-of-fit index, comparative fit index, incremental fit index, and Tucker-Lewis index all >0.9, root mean square error of approximation < 0.08, and root mean residual <0.5. The standardized regression coefficients of all paths in the CFA model were >0.45. The CR of the 4 dimensions was >0.7, and the AVE for all dimensions was >0.36. These findings suggest that the fit indices of the model generally meet the standards, indicating that the structural validity is relatively stable.^[34]^ It is widely acknowledged that the reliability coefficient of the overall scale should ideally exceed 0.8, the split-half reliability coefficient should also exceed 0.8, and the Cronbach α coefficient for individual subscales should be no <0.7.^[35]^ In this study, the reliability coefficient for the overall scale was 0.854, the Guttman split-half reliability coefficient was 0.811, and the Cronbach α coefficients for the 4 dimensions were 0.852, 0.822, 0.767, and 0.818, respectively. These findings suggest that the Chinese version of the SCI scale, as revised and adapted in this study, demonstrates robust internal consistency.

To explore the potential gender differences in sense of community, this study performed an independent samples t-test to compare the total SCI scores between male and female participants. The results showed that gender had no significant effect on the total SCI score. This finding is consistent with some previous studies reporting minimal overall gender differences in sense of community,^[8,36]^ although variations may emerge in specific aspects such as preferred participation formats, social interaction patterns, and access to community resources.

Community awareness, as a vital community resource, should be regarded as a key indicator of the success of community development efforts. The strength or weakness of community awareness must not be disregarded in the processes of community governance and development.^[37–39]^ In China, research on community awareness remains in its nascent stages, and the development of reliable and valid measurement tools is fundamental to advancing such research. This study involved the translation and revision of the English version of the SCI, followed by a comprehensive reliability and validity assessment of the Chinese version. The results indicated that all psychometric indices fell within acceptable thresholds, demonstrating that the scale is a valid and reliable tool for assessing community awareness among elderly residents in Chinese communities. The Chinese version of the SCI is concise, time-efficient, and user-friendly, embodying essential qualities of an effective assessment tool. By establishing a scientifically robust instrument for evaluating community awareness, this study provides valuable resources for community workers and researchers to better understand the levels of community awareness among the elderly. This, in turn, enables the implementation of targeted interventions, fostering greater community involvement among elderly individuals, and promoting the efficient and scientifically grounded advancement of community development.

### 4.1. Limitations

Although the revision of this scale has adhered to standardized guidelines for scale modification, it is imperative to systematically acknowledge several inherent limitations. First, given that China currently lacks a widely validated tool for assessing community awareness, the criterion-related validity of the scale remains unexamined. This indicates that the present scale should be regarded as an exploratory instrument introducing a novel measurement approach rather than a fully validated tool. Second, constrained by limitations in human, material, and financial resources, the study employed a convenience sampling method and was confined to elderly community residents in Bengbu City. Such methodological constraints may have introduced sampling bias, thereby restricting the generalizability of the findings. Expanding the research scope to encompass diverse populations and refining sampling strategies (e.g., adopting stratified or random sampling) would significantly enhance the external validity of subsequent investigations. Additionally, iterative refinement of scale items, coupled with supplementary psychometric evaluations and revisions, is warranted to improve the instrument’s precision and contextual applicability. Notably, a critical limitation pertains to the omission of self-reported disability status and functional impairments (e.g., mobility restrictions, sensory deficits) in the original demographic questionnaire. Given the well-documented influence of disability status on community belonging – including its effects on participatory opportunities, perceived social inclusion, and need fulfillment – future research should incorporate disability-related variables. This would enable a more comprehensive examination of older adults’ community experiences, facilitate the identification of potential disparities, and provide empirical support for developing targeted intervention strategies. Finally, while the sample size met the basic requirements for preliminary analysis, it remains inadequate for rigorous EFA and CFA. Larger sample sizes are essential to strengthen the robustness of factor structure validation and advance the scale’s psychometric credibility.

## 5. Conclusions

This study rigorously adhered to internationally recognized procedures for the adaptation of scales, obtaining both authorization and guidance from the original scale authors. Through expert consultations, cognitive interviews, and ongoing cultural adaptation, the scale was refined to ensure that all items were clear, unambiguous, and culturally appropriate within the Chinese context. The final version demonstrated robust reliability and validity and has been successfully introduced in China. The Chinese version of the Community Awareness Index Scale will serve as a reliable tool for assessing community awareness levels among elderly populations. It holds the potential to inform the development of targeted intervention strategies aimed at enhancing the quality of life and community belongingness among older adults.

## Acknowledgments

The authors would like to acknowledge the support of the elderly community members and community staff who participated in this study. In addition, we sincerely thank the editors for their hard work and the reviewers for their valuable suggestions on our manuscript.

## Author contributions

**Data curation:** Chengxiang Liu.

**Funding acquisition:** Ling Wang.

**Investigation:** Wei Xiang, Ping Sun.

**Methodology:** Wei Xiang.

**Writing – original draft:** Ling Wang.

**Writing – review & editing:** Qiao Xu, Hui Xie.
